# Identification of lncRNA and mRNA Expression Profile in Relapsed Graves’ Disease

**DOI:** 10.3389/fcell.2021.756560

**Published:** 2021-12-01

**Authors:** Qiuming Yao, Zhenyu Song, Bin Wang, Xi Jia, Ronghua Song, Jinan Zhang

**Affiliations:** ^1^ Department of Endocrinology, Shanghai University of Medicine and Health Sciences Affiliated Zhoupu Hospital, Shanghai, China; ^2^ Ovarian Cancer Program, Department of Gynaecologic Oncology, Zhongshan Hospital, Fudan University, Shanghai, China

**Keywords:** lncRNAs, relapsed GD, WGCNA, NONHSAT093153.2, NONHSAT209004.1

## Abstract

**Background:** Graves’ disease (GD) is a common autoimmune disease, and its pathogenesis is unclear. Studies have found that the occurrence of GD is related to the immune disorder caused by the interaction of genetic susceptibility and environmental factors. The CD4^+^ T cell subset is closely related to the immune disorder of GD. LncRNAs are RNA molecules with a length of more than 200 nt and are involved in a variety of autoimmune diseases. However, the roles of lncRNAs in recurrent GD are still elusive. The purpose of this study is to identify lncRNA and mRNA expression profile in relapsed Graves’ disease.

**Method:** CD4^+^ T cells from 12 recurrent GD and 8 healthy controls were collected for high-throughput sequencing. The gene-weighted co-expression network analysis (WGCNA) was used to construct the co-expression module relevant to recurrent GD, and the key genes in the module were verified by RT-PCR.

**Results:** There are 602 upregulated lncRNAs and 734 downregulated lncRNAs in CD4^+^ T cells in recurrent GD patients compared with the healthy controls. The module most relevant to GD recurrence was constructed using WGCNA, and the key genes in the module were verified by RT-PCR. We found that the expression of RPL8, OAS2, NFAT5, DROSHA, NONHSAT093153.2, NONHSAT118924.2, and NONHSAT209004.1 was significantly decreased in GD group (*p* < 0.001, *p* < 0.001, *p* < 0.01, *p* < 0.05, *p* < 0.001, *p* < 0.05, and *p* < 0.01, respectively).

**Conclusion:** LncRNAs are closely related to the recurrence of GD. For the first time, we constructed the expression profile of lncRNAs and mRNAs in CD4^+^ T cells in recurrent GD patients.

## Introduction

Graves’ disease, also known as toxic diffuse goiter, is characterized by the production of antibodies against thyroid stimulating hormone receptors (TRAb), leading to the hypertrophy and hyperfunction of the thyroid follicular cells (Morshed et al., 2012). GD is the most common cause of hyperthyroidism, and its incidence is about 0.5% (Brent, 2008). Hyperthyroidism caused by GD also increases the risk of atrial fibrillation, congestive heart failure, and miscarriage in pregnant women (Iddah and Macharia, 2013). However, the pathogenesis of GD is unclear, and its therapeutic effect is not satisfactory. At present, there are three main treatment methods for GD, including anti-thyroid drugs, radioactive iodine (RAI), and surgical resection. Each of the three methods has its own advantages and disadvantages. Compared with RAI and surgery, the disadvantage of anti-thyroid drugs mainly includes lower remission rate of hyperthyroidism and higher recurrence rate of patients with high TRAb titer. In addition, anti-thyroid drugs can also cause side effects such as skin rash, joint pain, agranulocytosis, and liver toxicity (Kotwal et al., 2018). Although radioactive iodine therapy has a higher cure rate, it has more chances of causing permanent hypothyroidism. Moreover, RAI is contraindicated in pregnant and lactating women and patients with active thyroid eye disease (Kotwal et al., 2018). Although surgical removal of the thyroid can quickly improve the symptoms of hyperthyroidism, patients need to take thyroid hormones throughout their lives, and the operation itself can cause a variety of complications such as hypoparathyroidism, recurrent laryngeal nerve injury, and neck hematoma. Therefore, it is urgent to further study the pathogenesis of GD and, on this basis, develop etiological treatment methods to reduce the serious harm of GD to public health.

Long non-coding RNAs (lncRNAs) are new members with more than 200 nucleotides in length of the non-coding RNAs (Caley et al., 2010). Although lncRNAs do not encode any protein products, they can regulate the gene expression at the transcriptional, post-transcriptional, and epigenetic level (Caley et al., 2010; Guttman et al., 2011). LncRNAs are also involved in functionally distinct biological and physiological processes such as chromatin remodeling, RNA junction, and protein transport (Mercer et al., 2009). Several studies have shown that lncRNAs are associated with autoimmune diseases, such as Crohn’s disease (Qiao et al., 2013), systemic lupus erythematosus (SLE) (Zhang Y. et al., 2021), and rheumatoid arthritis (Zhang J. et al., 2021), but the association of lncRNAs with relapsed GD remains unclear. Here, this study explored the potential roles of lncRNAs in relapsed GD.

## Materials and Methods

### Subjects

Forty-six relapsed GD patients and 33 age- and sex-matched normal healthy controls (NC) were enrolled from Zhoupu Hospital. Among them, 12 GD patients and 8 healthy controls were collected for lncRNA and mRNA sequencing, and the rest of subjects were recruited for the subsequent validation. Relapsed GD was diagnosed based on recurrence of clinical symptoms, elevated free triiodothyronine(FT3) or free thyroxine(FT4), suppressed thyroid-stimulating hormone(TSH), and positive anti-thyrotropin receptor antibody (TRAb) after a 12- to 18-month course of ATD treatment. We also detected the antibody against thyroglobulin (TGAb) or thyroid peroxidase (TPOAb) level of relapsed GD patients. Individuals without any acute or chronic autoimmune or allergic or infectious diseases or any acute or chronic visceral diseases were recruited as healthy controls or normal controls(NC). The study was approved by the Ethics Committee of Zhoupu Hospital. All subjects signed an informed consent form.

### CD4^+^ T Cell Isolation

Firstly, peripheral blood mononuclear cells (PBMCs) of all subjects were isolated from freshly collected venous blood by lymphocyte separation medium (Sigma-Aldrich) according to the manufacturer’s instruction. Then, the human CD4 Micro Beads (Miltenyi Biotec, Germany) was used to purificate CD4^+^ T cells from fresh PBMCs. The CD4^+^ T cells with a purity greater than 95% were used for further research. We calculated the purity of CD4^+^ T cells by flow cytometer (BD Biosciences, USA).

### Differentially Expressed Gene Screening

CD4^+^ T cells were isolated from PBMCs of 12 relapsed GD patients and 8 healthy controls. Then, we added 1 ml TRIzol to the CD4^+^ T cells. Total RNA was extracted from the CD4^+^ T cells using the TRIzol reagent (Takara) according to the manufacturer’s protocol. The samples were preserved at −80°C for further analysis. These samples were then subjected to mRNA and lncRNA-seq on the Illumina HiSeq platform following the standard procedures. The raw data were cleaned to obtain the reads with high quality. The clean reads with high quality were then aligned to the reference genome. Subsequently, the differentially expressed lncRNAs and mRNAs between GD patients and NC were screened in the expressing data using the “edeg” R package. The significantly changed genes with *p* < 0.05 and log2 fold change (FC)≥1 were considered as differentially expressed genes. Gene ontology (GO) enrichment and Kyoto encyclopedia of genes and genomes (KEGG) pathway analysis was conducted by R package.

### Construction of Co-Expression Module

We constructed the co-expression module of the differentially expressed lncRNAs and mRNAs (*p* < 0.05 and average expression level >1) between relapsed GD group and healthy controls by the R package “WGCNA”. We chose 5 as the soft-thresholding power, and 30 was chosen as the minimum number of modules.

### Hub Gene Identification

In the module-trait correlation analysis, the genes with gene significance greater than 0.4 and module group members (MM) greater than 0.9 were considered as hub genes, which are significantly associated with clinical phenotypes.

### Statistical Analysis

Software R3.5.3. was used to perform WGCNA analysis. The comparison between relapsed GD group and healthy controls was analyzed using non-parametric test. A *p* value less than 0.05 was considered statistically significant.

## Results

### The Expression Profile of lncRNAs and mRNAs in Relapsed GD CD4^+^ T Cells

To explore the crucial role of lncRNAs and mRNAs associated with the recurrence and development of GD, we performed RNA-Seq to detect the expression profile of lncRNAs and mRNAs in GD and NC group. Totally, we found that 1336 lncRNAs and 266 mRNAs were significantly differentially expressed between relapsed GD patients and healthy controls. Of the identified lncRNAs, 602 lncRNAs were significantly upregulated and 734 lncRNAs were significantly downregulated in the CD4^+^ T cells of GD patients.

**FIGURE 1:**
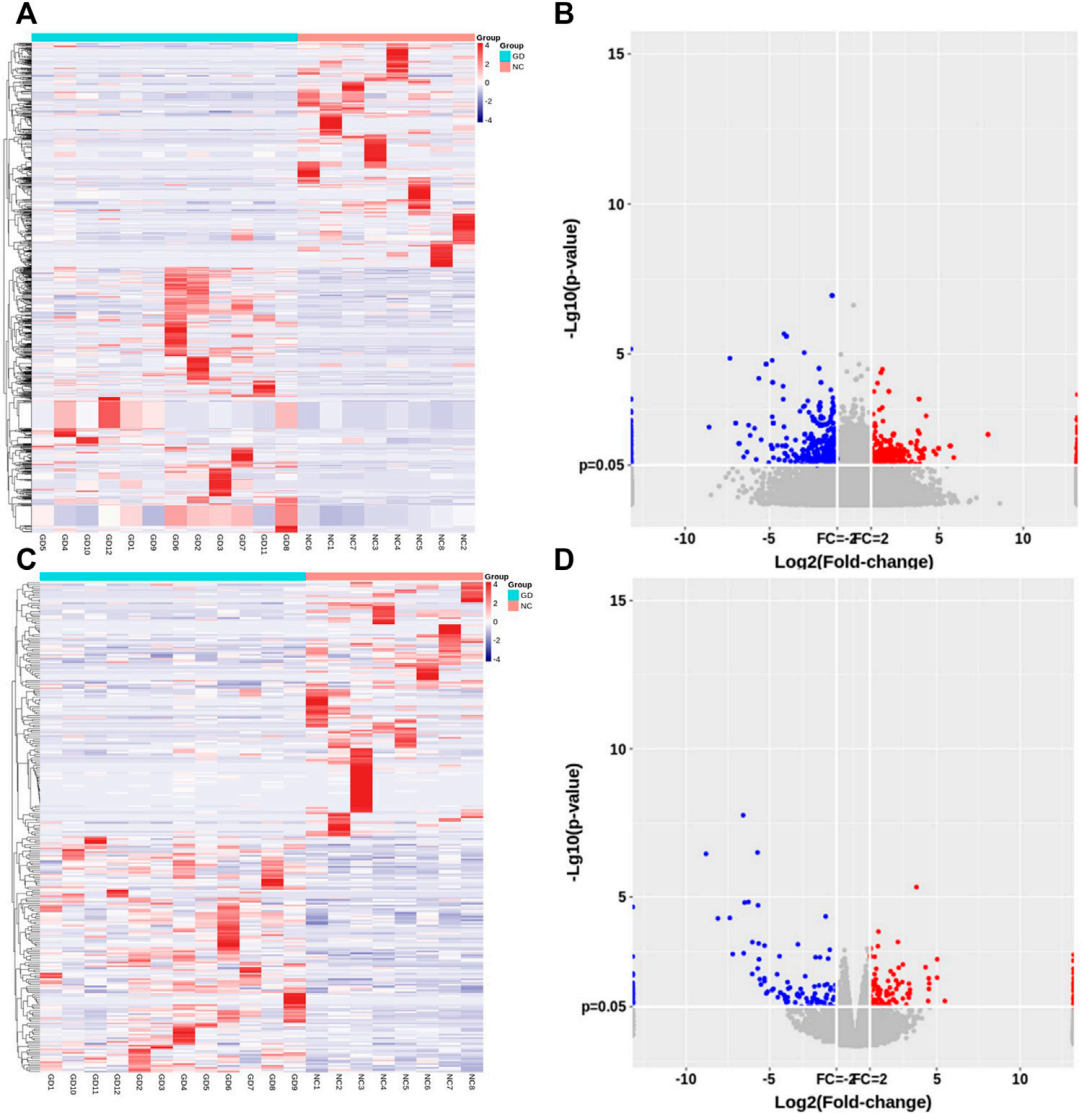
Expression profile of lncRNAs and mRNAs in CD4+ T cells of relapsed GD patients. **(A)** Hierarchical clustering of differentially expressed lncRNAs between GD group (*n* = 12) and normal controls (NC) (*n* = 8). **(B)** Volcano plots of lncRNA expression levels between two groups. **(C)** Hierarchical clustering of differentially expressed mRNAs between GD group (*n* = 12) and normal controls (NC) (*n* = 8). **(D)** Volcano plots of mRNA expression levels between two groups. Each column represents a sample, and each row indicates one gene. Red indicates those genes with relatively high expression level, and blue indicates those genes with relatively low expression level.

Of those detected mRNAs, 128 mRNAs were upregulated and 138 mRNAs were downregulated in CD4^+^ T cells of GD patients. Hierarchical cluster analyses displayed lncRNA and mRNA expression profile in two groups (Figures 1A,C). Volcano plot analyses were also performed to visualize the differentially expressed lncRNAs and mRNAs (Figures 1B,D).

### GO Analysis and Pathway Analysis

**FIGURE 2:**
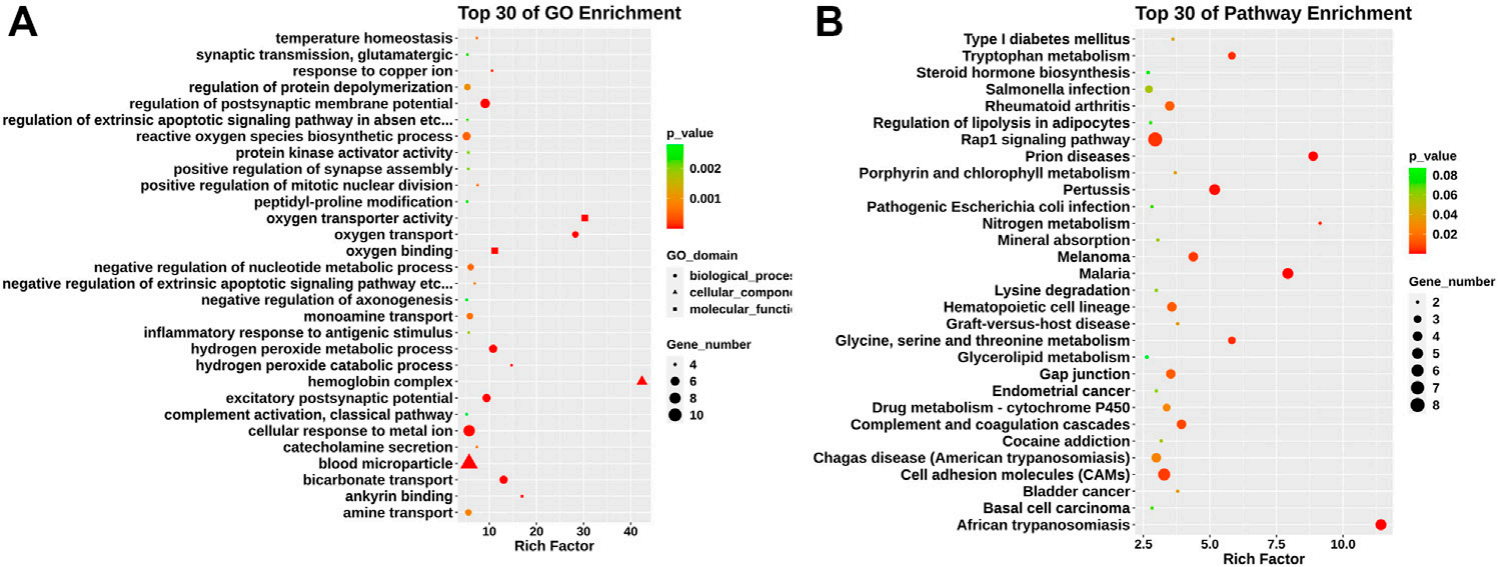
GO and KEGG pathway analysis in relapsed GD CD4^+^ T cells. **(A)** GO analysis of differentially expressed genes between GD group and contols. According to biological process (circle), cellular component (triangle), and molecular function (square). **(B)** KEGG pathway analysis for differentially expressed mRNAs.

We conducted Gene ontology (GO) and KEGG pathway enrichment analyses to further explore the function of those differentially expressed genes. The GO analysis found that differentially expressed genes identified were mainly enriched in hemoglobin complex, oxygen transporter activity, excitatory postsynaptic potential, and positive regulation of mitotic nuclear division (Figure 2A). KEGG pathway analysis revealed that those genes were mainly enriched in glycine, serine, and threonine metabolism, complement and coagulation cascades, and cell adhesion molecules (CAMs) (Figure 2B).

### WGCNA Analysis

**FIGURE 3:**
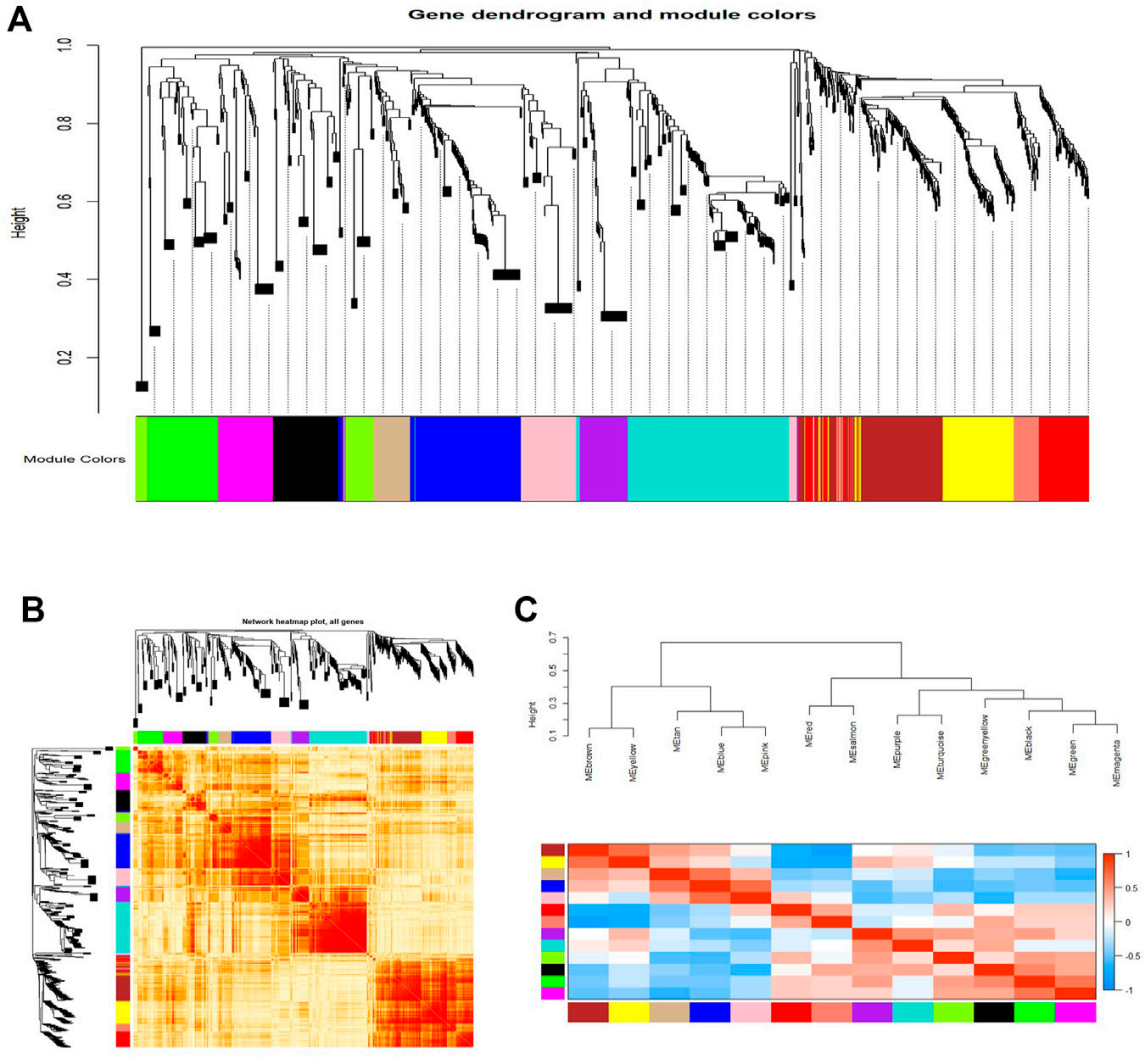
WGCNA revealed gene co-expression modules in the CD4+ T cells of relapsed GD patients. **(A)** Clustering dendrograms of mRNAs and lncRNAs. Each color represents a co-expression module. **(B)** Network heatmap plot in the co-expression modules. **(C)** Eigengene dendrogram and eigengene adjacency heatmap.

As shown in Figure 3, we constructed a total of 13 co-expression modules by WGCNA analysis (Figure 3A). Moreover, these constructed modules were independent of each other (Figure 3B). Figure 3C shows an eigengene dendrogram and adjacency heatmap.

**FIGURE 4:**
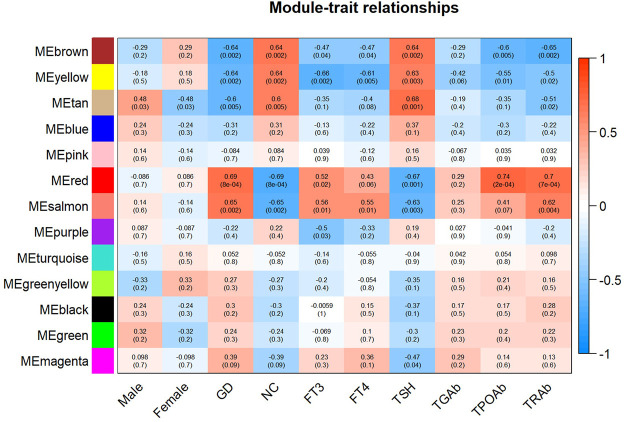
Heatmap of the correlation between each coexpression module and relapsed GD and different phenotypes. The phenotypes mainly include sex, FT3, FT4, TSH, TGAb, TPOAb, and TRAb. (The correlation coefficient and corresponding *p* value were shown in each cell.)

**FIGURE 5:**
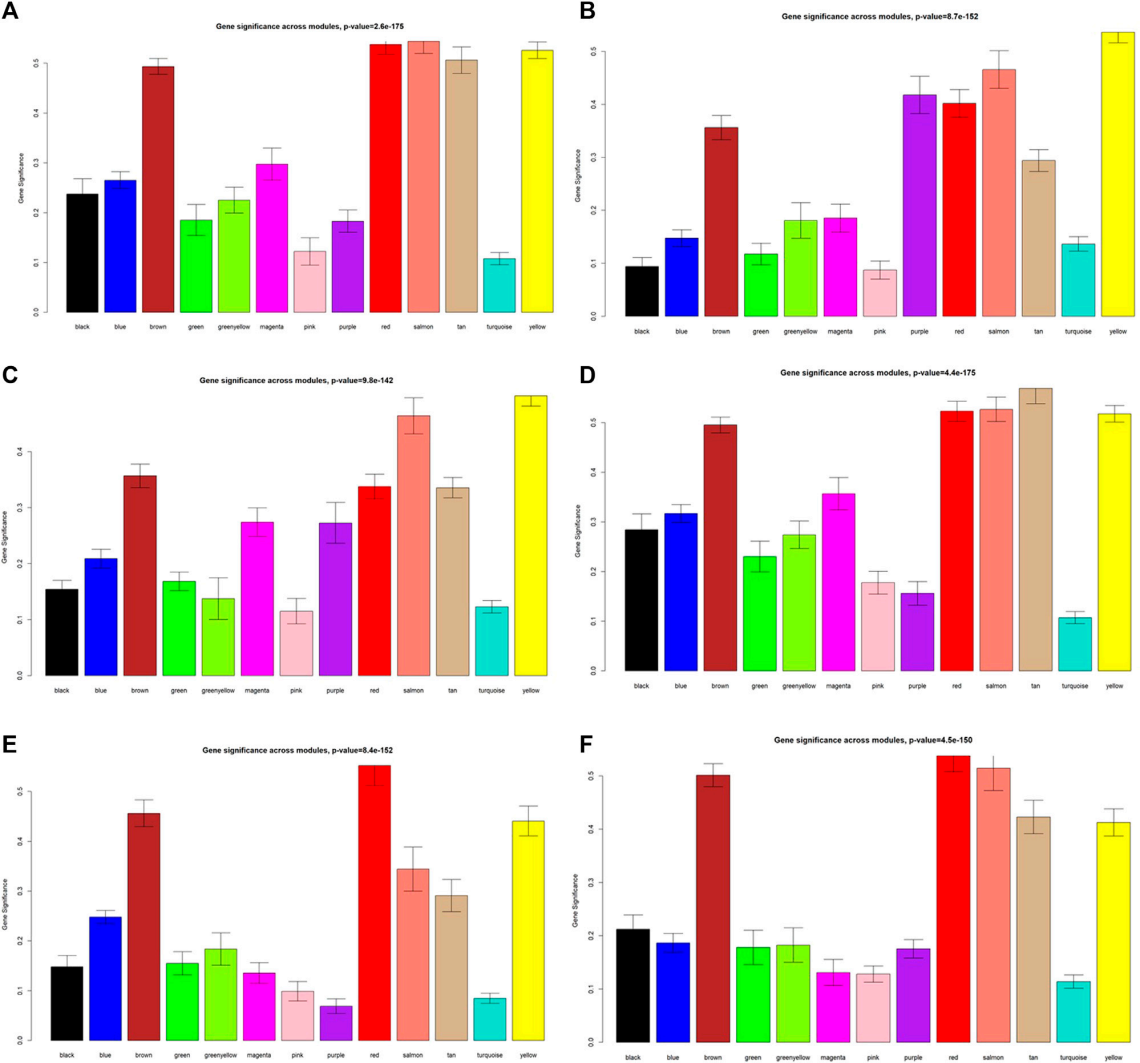
Module significance values of co-expression modules related to different phenotypes. **(A)** GD; **(B)** FT3; **(C)** FT4; **(D)**, TSH; **(E)** TPOAb; **(F)** TRAb. Each color indicated one coexpression module.

**FIGURE 6:**
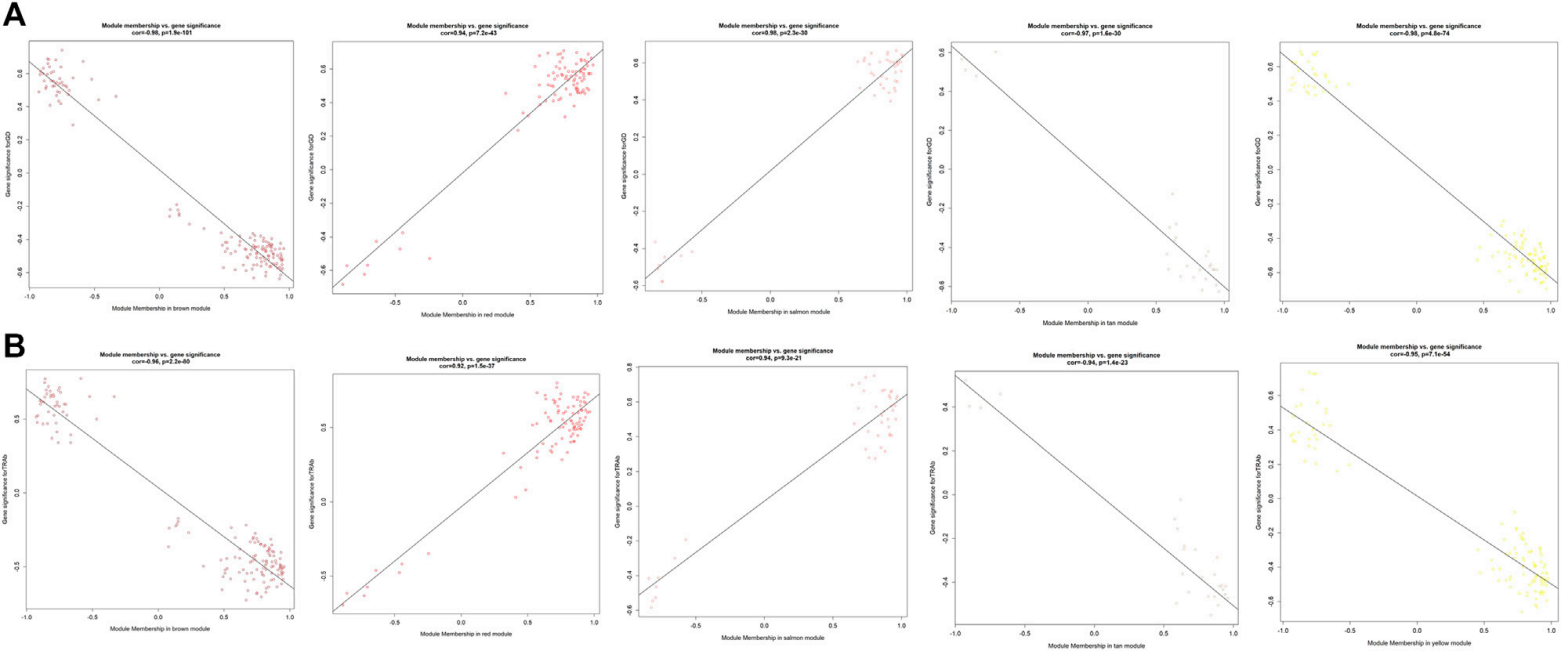
The scatterplots of different modules highly related to GD and TRAb. **(A)** GD; **(B)** TRAb. The correlation coefficient and *p* value of module membership vs. gene significance were shown.

As displayed in Figure 4, module-trait correlations showed that five modules were related to GD, including red, salmon, brown, yellow, and tan module. Interestingly, all these modules are also related to TSH and TRAb. While brown, yellow, red, salmon, and purple modules were associated with FT3, three modules including brown, yellow, and salmon were related to FT4. The brown, yellow, and red modules were associated with TPOAb. Figure 5 shows module significance values of co-expression modules associated with each phenotype, including GD (Figure 5A), FT3 (Figure 5B), FT4 (Figure 5C), TSH (Figure 5D), TPOAb (Figure 5E) and TRAb (Figure 5F). Figure 6 shows the scatterplots of gene significance for GD (Figure 6A) and TRAb (Figure 6B) vs. MM in different modules.

### Functional Annotation of Key Co-Expression Modules

**FIGURE 7:**
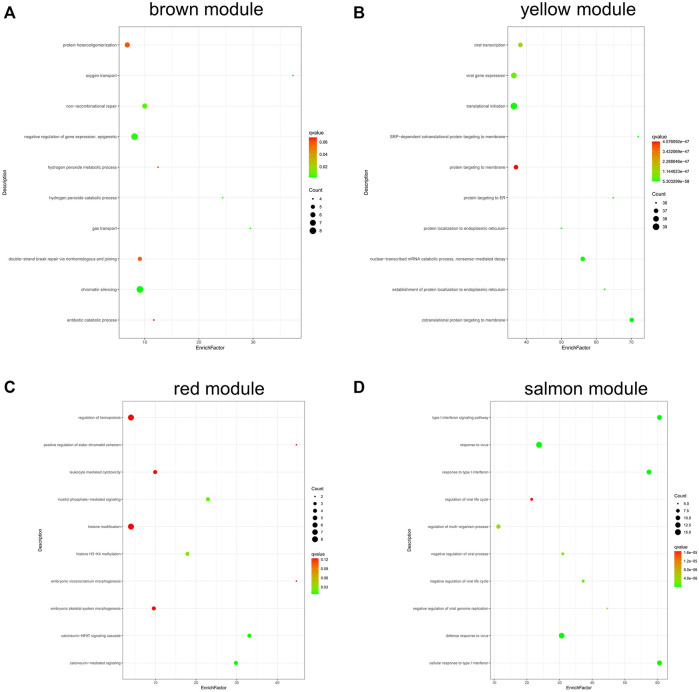
GO analysis of genes in coexpression modules associated with relapsed GD. **(A)** Brown module; **(B)** Yellow module; **(C)** Red module; **(D)** Salmon module.

Figure 7 shows the results of GO analysis about genes in different module. Genes in brown module were mainly enriched in negative regulation of gene expression and epigenetic and chromatin silencing (Figure 7A); genes in yellow module were enriched in viral transcription, viral gene expression, and nuclear-transcribed mRNA catabolic process (Figure 7B); genes in red module were mainly enriched in inositol phosphate-mediated signaling, histone H3-K4 methylation, calcineurin-NFAT signaling cascade, and calcineurin-mediated signaling (Figure 7C); the salmon module was mainly enriched in type I interferon signaling pathway, response to virus, etc (Figure 7D).

**FIGURE 8:**
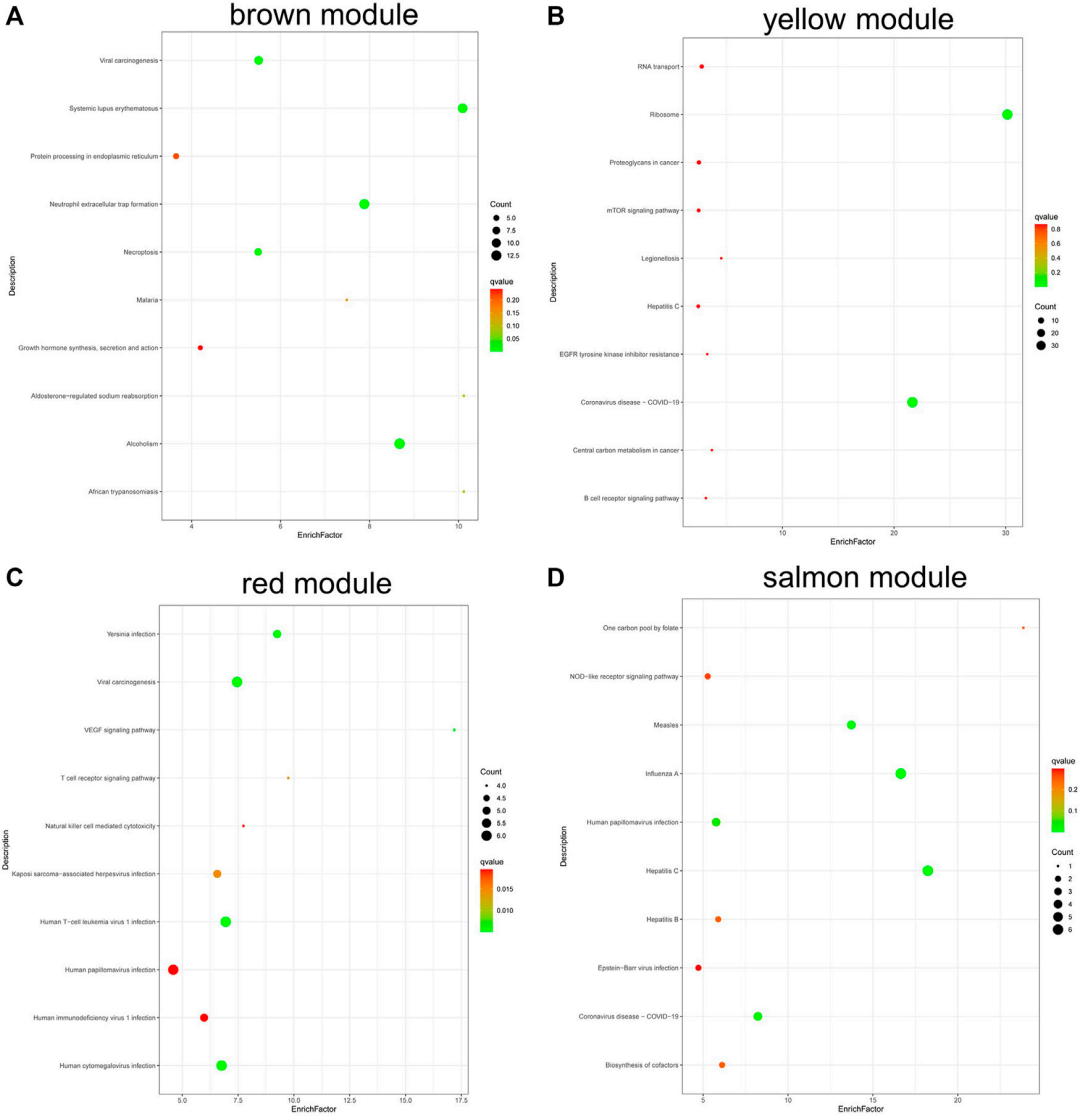
KEGG pathway analysis of genes in coexpression modules associated with relapsed GD. **(A)** Brown module; **(B)** Yellow module; **(C)** Red module; **(D)** Salmon module.

KEGG analysis (Figure 8) found that the genes in brown module were involved in alcoholism, systemic lupus erythematosus, and neutrophil extracellular trap formation (Figure 8A); genes in yellow module were involved in ribosome, coronavirus disease—COVID-19, RNA transport, etc. (Figure 8B). The genes in red module were involved in VEGF signaling pathway, viral carcinogenesis, and Yersinia infection (Figure 8C), and the genes in salmon module were involved in hepatitis C, influenza A, measles, human papillomavirus infection, and biosynthesis of cofactors (Figure 8D).

### Validation of Hub Genes

**FIGURE 9:**
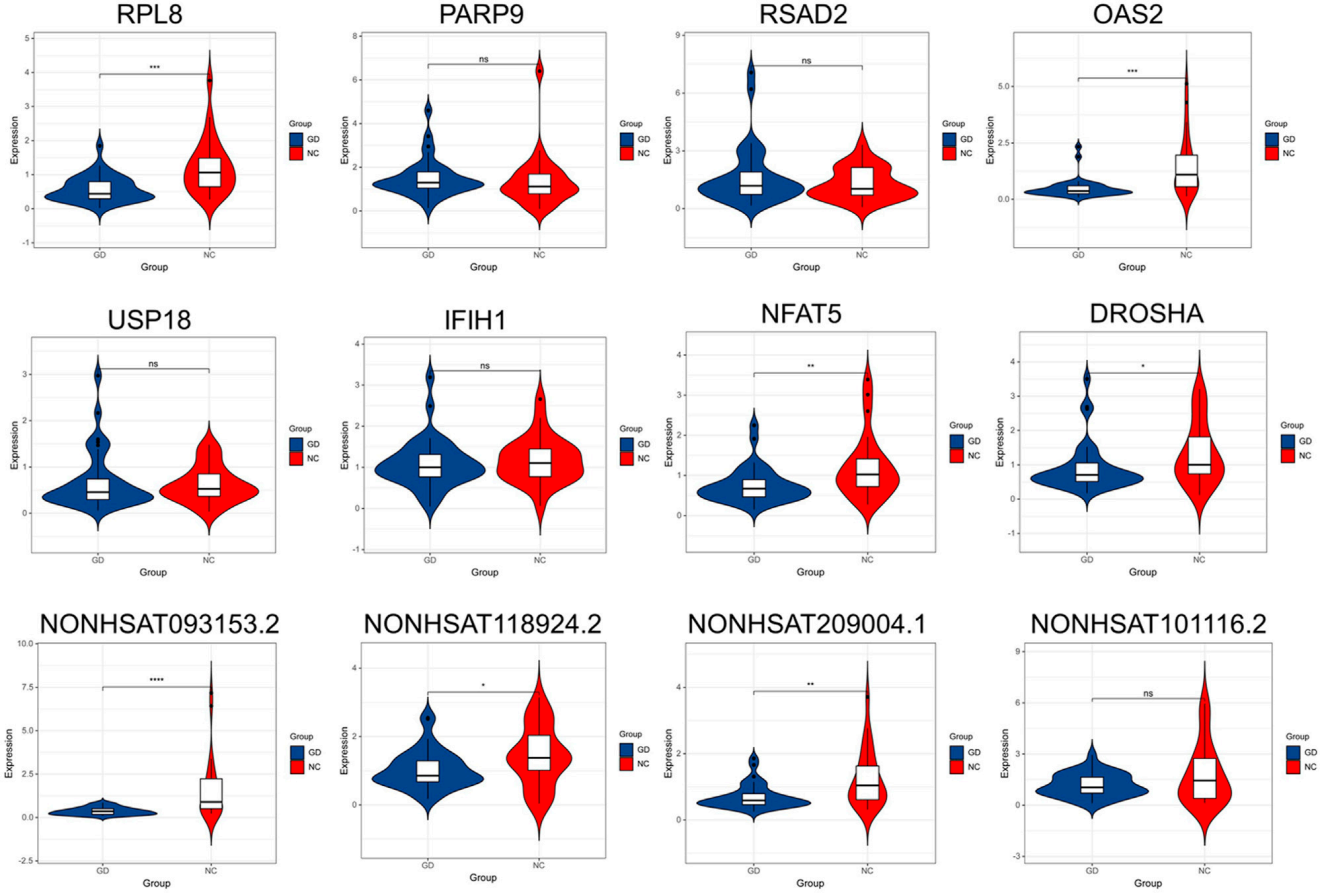
Validation of key lncRNAs and mRNAs by qRT-PCR. The expression of RPL8, OAS2, NFAT5, DROSHA, NONHSAT093153.2, NONHSAT118924.2, and NONHSAT209004.1 was significantly decreased in GD group (*p* < 0.001, *p* < 0.001, *p* < 0.01, *p* < 0.05, *p* < 0.001, *p* < 0.05, and *p* < 0.01, respectively). No significant difference was found in the expression level of PARP9, RSAD2, OAS2, USP18 A, and NONHSAT101116.2 between GD and normal control (NC) groups. ∗*p* < 0.05, ∗∗*p* < 0.01, ∗∗∗ or ∗∗∗∗ *p* < 0.001, ns, not significant.

The gene with gene significance value greater than 0.4 and module membership greater than 0.9 is considered as the hub gene. We verified the expression of eight mRNAs and six lncRNAs of interest by PCR, including RPL8 in brown module, PARP9, RSAD2, OAS2, USP18, and IFIH1 in salmon module, NFAT5 and DROSHA in red module, and NONHSAT093153.2, NONHSAT209004.1, NONHSAT101116.2, NONHSAT161865.1, NONHSAT118924.2, and NONHSAT077537.2 in tan module. As shown in Figure 9, our results showed that the expression of RPL8, OAS2, NFAT5, DROSHA, NONHSAT093153.2, NONHSAT118924.2, and NONHSAT209004.1 was significantly decreased in GD group (*p* < 0.001, *p* < 0.001, *p* < 0.01, *p* < 0.05, *p* < 0.001, *p* < 0.05, and *p* < 0.01, respectively). However, there was no significant difference in the expression level of PARP9, RSAD2, OAS2, USP18 A, NONHSAT101116.2, NONHSAT077537.2 (data not shown), and NONHSAT161865.1 (data not shown) between the two groups (all *p* > 0.05.)

## Discussion

Considering the high recurrence rate of GD after treatment, research on clarifying the pathogenesis of GD is an important but challenging task. The lymphocyte infiltration results in the destruction of thyroid tissues and amplifies the extent of autoimmune response. Among the lymphocytes, CD4^+^ T cells play an important role in the pathogenesis of GD, which mainly includes Th1, Th2, Th17, Th22, Tfh cells, and Treg. Emerging studies have shown that the imbalance between Th1 and Th2 cells leads to GD (Zemmour et al., 2017). Recent studies have revealed that the abnormal expression of Th17 (Su et al., 2020; Zake et al., 2021), Th22 (Peng et al., 2013; Vitales-Noyola et al., 2017), Tfh cells (Liu et al., 2018), and Treg (Chen et al., 2021a) is associated with GD pathogenesis. The above findings illustrated that the dysfunction of CD4^+^ T cells plays a vital role in the development of GD. Nevertheless, the underlying mechanisms of CD4^+^ T cell dysfunction need to be further clarified. In the present study, we generated a signature profile of numerous lncRNAs and mRNAs in CD4^+^ T cells of relapsed GD patients compared with healthy controls by high-throughput sequencing technologies.

We obtained a total of 13 co-expression modules by WGCNA analysis. Among them, five modules including brown, yellow, tan, red, and salmon module were the main modules involved in GD, containing 144, 105, 49, 90, and 43 genes, respectively.

Currently, widely used GO analysis is very powerful in classifying various biological entities into functional related groups (Rue-Albrecht et al., 2016). In the present study, we also used GO analysis to study the biological functions of genes in the five modules.

Our results showed that the genes in brown module were mainly enriched in negative regulation of gene expression and epigenetic and chromatin silencing; the genes in red module were mainly enriched in inositol phosphate-mediated signaling, histone H3-K4 methylation, calcineurin-NFAT signaling cascade, and calcineurin-mediated signaling; genes in salmon module were mainly enriched in type I interferon signaling pathway, response to virus, etc. The genes in yellow module were enriched in viral transcription, viral gene expression, etc. These findings suggest that multiple biological processes are involved in the pathogenesis of relapsed GD.

Among the identified hub genes, we found the expression of three lncRNAs (NONHSAT093153.2, NONHSAT118924.2, and NONHSAT209004.1) and four mRNAs (RPL8, OAS2, NFAT5, and DROSHA) were significantly downregulated in the relapsed GD patients, suggesting that these genes are involved in the occurrence of recurrent GD.

NONHSAT093153.2, NONHSAT118924.2, and NONHSAT209004.1 were firstly investigated in the relapsed GD patients. RPL8, a member of ribosomal proteins, is a component of the 60S ribosomal subunit in eucaryotic cells (Sun et al., 2015). It has been reported that RPL8 was related to multiple sclerosis (MS) and was a potential biomarker of MS(Chen et al., 2021b). RPL8 has not been reported in GD, and our study suggests that RPL8 was significantly decreased in GD and is worthy of further study. NFAT5 is a member of the Rel family of transcriptional factors (Lopez-Rodriguez et al., 1999). Recent emerging studies have reported the role of NFAT5 in the development and activation of macrophages and T cells (Lee et al., 2019). NFAT5 can induce the activation of pathogenic pro-inflammatory macrophages and pathogenic Th17 cells (Choi et al., 2016; Aramburu and López-Rodríguez, 2019). Numerous studies found that increased expression of NFAT5 was involved in inflammatory and autoimmune diseases (Choi et al., 2017; Choi et al., 2018). OAS2 is a potential new sensitive biomarker, which can predict the activity and severity of psoriasis, and can evaluate the clinical treatment efficacy (Zhou et al., 2020). OAS2 can also be considered as biomarker gene for systemic lupus erythematosus (SLE) diagnosis (Fang et al., 2021). OAS family genes including OAS2 were revealed to be closely related to lupus nephritis (Cao et al., 2020). Drosha is RNase III enzyme necessary for most miRNA biogenesis. Study has found that the Drosha polymorphism was associated with GD development (Saeki et al., 2016). Our results showed that Drosha expression was significantly decreased in relapsed GD patients.

Although IFIH1, RSAD2, and PARP9 were found to be associated with a variety of autoimmune or inflammatory disease development, such as SLE, RA, Sjögren’s syndrome (SS), type 1 diabetes (T1D), and AITD (Frommer and Kahaly, 2021; Zedan et al., 2021), we did not find that these genes were differentially expressed between the recurrent GD group and the normal group. Ubiquitin-specific peptidase 18 (USP18) plays a crucial role in the development of Th17 cells and can regulate the differentiation and function of Treg cells (Yang et al., 2021). In our study, no significant difference was found in the USP18 expression level between relapsed GD group and NC.

The present study also has some limitations. Firstly, we did not further explore the molecular mechanism of the hub genes in relapsed GD. Secondly, the number of samples we recruited to verify gene expression was too small, because blood samples from patients with recurrent GD are very difficult to collect. Thus, the hub gene expression and the potential role of them in GD still need to be further investigated and validated in more samples.

In summary, this study is the first to explore the coexpression gene networks including lncRNAs and mRNAs related to relapsed GD through WGCNA analysis with large sample size. Our study mainly finds involvement of the key gene co-expression modules, functional biological pathways, and hub genes in the development of relapsed GD. Although the potential mechanism of functional pathways and hub genes in relapsed GD still needs to be further investigated, these initial and innovative findings provide new insights into the pathogenesis of relapsed GD undoubtedly.

## Data Availability

The datasets presented in this study can be found in online repositories. The names of the repository/repositories and accession number(s) can be found below: NCBI [accession: PRJNA763124].
